# Hierarchical Bayesian modelling of disease progression to inform clinical trial design in centronuclear myopathy

**DOI:** 10.1186/s13023-020-01663-7

**Published:** 2021-01-06

**Authors:** Eve Fouarge, Arnaud Monseur, Bruno Boulanger, Mélanie Annoussamy, Andreea M. Seferian, Silvana De Lucia, Charlotte Lilien, Leen Thielemans, Khazal Paradis, Belinda S. Cowling, Chris Freitag, Bradley P. Carlin, Laurent Servais, the NatHis-MTM Study Group, Teresa Gidaro, Elena Gargaun, Virginie Chê, Ulrike Schara, Andrea Gangfuß, Adele D’Amico, James J. Dowling, Basil T. Darras, Aurore Daron, Arturo Hernandez, Capucine de Lattre, Jean-Michel Arnal, Michèle Mayer, Jean-Marie Cuisset, Carole Vuillerot, Stéphanie Fontaine, Rémi Bellance, Valérie Biancalana, Ana Buj-Bello, Jean-Yves Hogrel, Hal Landy, Kimberly Amburgey, Barbara Andres, Enrico Bertini, Ruxandra Cardas, Séverine Denis, Dominique Duchêne, Virginie Latournerie, Nacera Reguiba, Etsuko Tsuchiya, Carina Wallgren-Pettersson

**Affiliations:** 1 Division of Child Neurology, Centre de Référence Des Maladies Neuromusculaires, Department of Paediatrics, University Hospital of Liège and University of Liège, Liège, Belgium; 2 Pharmalex Belgium, Mont-Saint-Guibert, Belgium; 3 Institute I-Motion, Hôpital Armand Trousseau, Paris, France; 4 Sysnav, Vernon, France; 5 MDUK Oxford Neuromuscular Centre, Department of Paediatrics, University of Oxford, Oxford, UK; 6 Dynacure, 67400 Illkirch, France; 7 2 Bridge, Rodendijk 60/X, 2980 Zoersel, Belgium; 8 Paradis Consultancy SAS, 06570 Saint-Paul-de-Vence, France; 9 PharmaLex US, Burlington, MA USA; 10 Department of Paediatrics, Level 2, John Radcliffe Hospital, Headley Way, Headington, OX3 9DU Oxford UK

**Keywords:** Bayesian analysis, Centronuclear myopathy, Complex innovative clinical trial design, Disease progression model, Natural history data

## Abstract

**Background:**

Centronuclear myopathies are severe rare congenital diseases. The clinical variability and genetic heterogeneity of these myopathies result in major challenges in clinical trial design. Alternative strategies to large placebo-controlled trials that have been used in other rare diseases (e.g., the use of surrogate markers or of historical controls) have limitations that Bayesian statistics may address. Here we present a Bayesian model that uses each patient’s own natural history study data to predict progression in the absence of treatment. This prospective multicentre natural history evaluated 4-year follow-up data from 59 patients carrying mutations in the *MTM1* or *DNM2* genes.

**Methods:**

Our approach focused on evaluation of forced expiratory volume in 1 s (FEV1) in 6- to 18-year-old children. A patient was defined as a responder if an improvement was observed after treatment and the predictive probability of such improvement in absence of intervention was less than 0.01. An FEV1 response was considered clinically relevant if it corresponded to an increase of more than 8%.

**Results:**

The key endpoint of a clinical trial using this model is the rate of response. The power of the study is based on the posterior probability that the rate of response observed is greater than the rate of response that would be observed in the absence of treatment predicted based on the individual patient’s previous natural history. In order to appropriately control for Type 1 error, the threshold probability by which the difference in response rates exceeds zero was adapted to 91%, ensuring a 5% overall Type 1 error rate for the trial.

**Conclusions:**

Bayesian statistical analysis of natural history data allowed us to reliably simulate the evolution of symptoms for individual patients over time and to probabilistically compare these simulated trajectories to actual observed post-treatment outcomes. The proposed model adequately predicted the natural evolution of patients over the duration of the study and will facilitate a sufficiently powerful trial design that can cope with the disease’s rarity. Further research and ongoing dialog with regulatory authorities are needed to allow for more applications of Bayesian statistics in orphan disease research.

## Background

In the past two decades, there has been a dramatic increase in development of therapies and therefore in clinical trials of patients with rare diseases. The number of publications in this area rose by more than five-fold between the years 2003 and 2018 [1]. Furthermore, the number of molecules in clinical development increased by five-fold between 2013 and 2018 [2]. However, there are many challenges related to conducting clinical studies on rare diseases, such as heterogeneity in pathophysiology or clinical presentation, the overdue window of treatment opportunity in some conditions, and the difficulties in conducting adequately powered trials in rare diseases where the number of patients is low. The challenge of recruiting a large population needed to conduct a placebo-controlled study may lead to an enlargement of inclusion criteria and thus an increase of the heterogeneity of the population.

In the context of neuromuscular diseases, several strategies have been used to gain regulatory approval of orphan drugs without conducting large, placebo-controlled trials. These include the use of surrogate markers such as dystrophin in the clinical trial of eteplirsen in patients with Duchenne muscular dystrophy [3] and globotriaosylceramide in the clinical trial of agalsidase beta in patients with Fabry disease [4]. Historical controls were used during clinical testing of onasemnogene abeparvovec-xioi in spinal muscular atrophy [5] and alglucosidase alfa in Pompe disease [6], and a priori-designed natural history studies were used in the clinical evaluation of risdiplam in patients with spinal muscular atrophy type 2 [7]. However, these strategies have limitations.

Surrogate markers (also called surrogate endpoints) are substitute outcomes that are studied when a desired primary clinical endpoint such as overall survival takes too long to observe or is ethically unjustifiable [8]. In the cases of eteplirsen and golodirsen, two antisense oligonucleotides that induce skipping of exons 51 and 53, respectively, of the *dystrophin* pre-mRNA, the surrogate nonclinical endpoint was the induction of truncated dystrophin production [9, 10]. The U.S. Food and Drug Administration (FDA) approved the drugs in 2016 and 2019, respectively, under the accelerated approval program after concluding that enhanced dystrophin expression was reasonably likely to result in clinical benefit. Neither of these two drugs were, however, approved by the European Medicines Agency (EMA), which requires that a surrogate endpoint first be validated by showing a correlation between the surrogate endpoint and a clinical benefit [11]. Furthermore, not all diseases have sensible surrogate endpoints and, in some cases, surrogate endpoints have been called into question based on later studies. In the early stages of the HIV/AIDS epidemic, change in CD4^+^ T cell count was used as a surrogate marker in several trials, but this measure was eventually shown to be only weakly associated with survival [12].

In the examples of historical cohorts cited above, survival and need for ventilation support of patients with Pompe disease treated with alglucosidase alfa [13], and motor function scores and motor milestones in spinal muscular atrophy patients treated with onasemnogene abeparvovec-xioi [14] were compared to the natural history of the diseases. To show the efficacy of a drug in a retrospective cohort study, however, the drug’s effects must be substantial, since other factors, such as a difference in standard of care or a placebo effect, may also explain differences between treated patients and historical cohorts.

The FDA has acknowledged the fact that rare disease clinical trials necessitate innovative designs that make use of, for example, external control patients and information on disease progression from natural history studies to improve the analytical model [15]. Accordingly, an alternative strategy is the use of Bayesian statistics [16]. Bayesian methods permit the reallocation of the probability of an explanation following the acquisition of new data related to a previously selected set of possible explanations. The Bayesian approach permits the borrowing of strength from additional information sources including, for example, historical controls from earlier randomized studies, data from disease registries, natural history studies and other nonrandomized sources, and expert medical opinion. Although non-Bayesian methods may also be used to compare treated patients with existing dataset or to follow evolution of disease at a population level, the Bayesian framework is more convenient for deriving prediction intervals with complex stochastic processes such as the beta distribution at both the population and individual levels [16]. The result is a gain in study power and corresponding reduction in sample size needed. There is also a corresponding modest increase in overall Type I error (i.e., the false positive rate) that is typically estimated and controlled via pre-trial simulation studies. Bayesian methods have been discussed by both the EMA [17, 18] and the FDA [15, 19–21] with the conclusion that Bayesian methods offer a statistically acceptable approach, especially in rare and paediatric disease settings.

In this article, we leverage Bayesian methods to utilize a trial enrolee’s own personal natural history study (NHS) data to supplement recorded trial outcomes. Our NHS was a prospective international study on X-linked and autosomal dominant centronuclear myopathy, which followed Good Clinical Practice and systematic source data verification. Patient forced expiratory volume in 1 s (FEV1), assessed according to EU and US recommendations, and time on ventilator were recorded at every visit [22]. The Bayesian model reduced the necessary sample size while controlling overall Type I error. Importantly, the model is *hierarchical,* in that it can estimate both individual and overall population-level outcome trajectories and treatment effects.

We applied our hierarchical Bayesian modelling approach to the field of centronuclear myopathies (CNMs). This is a group of rare congenital myopathies with a highly variable clinical presentation and substantial genetic heterogeneity. Because of rarity and high variability, the incidence of centronuclear myopathies is not well known. However, the incidence of its most frequent and severe form, X-linked myotubular myopathy (XLMTM), is approximately 1 in 50 000 new-born males [23]. The diagnosis is suggested by the central position of nuclei in muscle biopsies and clinical features. X-linked, autosomal recessive and autosomal dominant forms of CNM have been identified. The X-linked form is usually more severe, and symptoms are present at birth, yet a broad clinical heterogeneity is observed. The main causal mutations are distributed throughout the genes encoding myotubularin (*MTM1*) for XLMTM (OMIM: 310400) [24], dynamin 2 (*DNM2*) [25] and amphiphysin 2 (*BIN1*) [26] for the autosomal dominant form (OMIM: 160150), and amphiphysin 2 (*BIN1*) [27] for the autosomal recessive form (OMIM: 255200). The clinical traits of CNM include hypotonia, external ophthalmoplegia, and respiratory deficiency, which can be severe and life-threatening in the XLMTM congenital form [22, 28]. Patients who survive beyond the neonatal period live with a high disease burden: a majority require the use of a wheelchair, feeding tube, and ventilation support. Additionally, respiratory function is also altered in patients who do not need ventilator support and respiratory complications are the most frequent cause of death [29, 30]. Despite their rarity and their heterogeneous genotype and phenotype, CNMs are currently the targets of several clinical and pre-clinical development efforts that make them a paradigm for the need of alternative statistical strategies in clinical trials [31].

## Methods

### Bayesian disease progression model

In the particular setting of rare diseases, Bayesian models can be used to predict disease progression based on endpoints envisaged in the considered trial. The steps of this strategy can be summarized as follows:

1. **Bayesian disease progression model** A hierarchical Bayesian disease progression model based on NHS data is developed and documented. The hierarchical model (sometimes called a mixed-effects model) permits estimation of trajectories for each patient as well as evaluation of patient-to-patient variability and heterogeneity in these trajectories. Mathematically, the observed response $y_{ij}$, scaled between 0 and 1, is defined as:
  $$y_{ij} \sim Beta\left( {a_{ij} ,b_{ij} } \right)$$
  where the beta distribution is defined by the parameters $a_{ij} \,{\text{and}}\,b_{ij}$ (where *i* indicates the subject and *j* the time), which are defined, respectively, as the mean $\mu_{ij}$ and “the sample size” $\nu$ of the distribution as follows:
  $$\begin{aligned} a_{ij} & = \mu_{ij} *\nu_{ij} \\ b_{ij} & = \left( {1 - \mu_{ij} } \right)*\nu_{ij} \\ \end{aligned}$$
  The parameter $\nu_{ij}$ is estimated from the data, and the mean $\mu_{ij}$ is defined as a mixed model with logit-link function:
  $$\begin{aligned} \chi_{ij} & = \alpha_{i} + \beta_{i} *\left( {T + t_{j} } \right) \\ \mu_{ij} & = \frac{1}{{1 + exp\left( { - \chi_{ij} } \right)}} \\ \end{aligned}$$
  where *T* is a constant to centre time. The variability in the model can be derived using properties from the beta distribution. The evolution of the mean is linear on the logistic scale. This implies that except near the boundary the evolution of the response is approximately linear. This is meaningful for short periods of time in the context of CNM-related disease as supported by the data.
  On a long time scale, the evolution of patients is probably not linear, but when reduced to a trial compatible-time scale of 6 months or 1 year, linearity is a reasonable expectation. A similar assumption of linearity is regularly accepted, for instance, when evaluating Duchenne patients with the 6-min-walk-distance when the time window is on the order of a year even though this is a measure with a non-linear inverse U-shaped evolution over the life-time course [32].
2. **Individual prediction** Combining the NHS data with the run-in data from patients enrolled but not included in the NHS allows the derivation of individual predictive distributions of the endpoint or endpoints over time after treatment administration. Only data pre-treatment and from the NHS are considered. Stated differently, no data gathered post-administration is included in the model.
3. **Identifying responders** The joint predictive probability of improvement (increase or decrease over time depending on the endpoint measured) is computed for each patient. If the joint predictive probability of the patient’s observed improvement is smaller than or equal to 0.01, then a patient is declared to be a responder. We adopted this predictive probability-based definition of responder since (a) there is little consensus on what constitutes a clinically relevant change and (b) the assessment of the primary endpoint (FEV1 versus time on ventilator) will differ from patient to patient depending on age and disability level.
4. **Control of trial Type I error** Using this definition of responder, the predictive distribution of the rate of response is simulated under the null hypothesis of no treatment effect. A statistical significance threshold value is then determined to guarantee an overall Type I error of less than or equal to 5%.

The same model was applied to some potential primary and secondary endpoints (FEV1, FVC, CHOP-INTEND, MFM20, MFM32, and time on respirator) by age class and by genotype. The model used is a hierarchical Bayesian model with beta distribution likelihood and a logit link function. Random effects for each patient were used to model the differences in level and progression of the disease. Since all responses are scores bounded by an upper and lower value, the beta distribution is appropriate for the modelling, as it is readily scaled to be bounded between any two real numbers. The beta thus ensures that predictions do not fall outside the possible score range. The model was readily fit using Proc MCMC in SAS 9.4. Diffuse and non-informative prior distributions were used for all unknown parameters so that the observed data drove our model fits.

### NHS population

The NHS population consisted of 59 patients recruited in Europe [22]. Of the 59 patients, 15 have a *DNM2* mutation and 44 patients have a mutation in the *MTM1* gene (Table 1). These patients had been evaluated every 3 months under 2 years of age, every 6 months between 2 and 6 years of age, and, for patients older than 6, at 6 months and 12 months after enrolment and then once a year. The study was prospective, and patients were mostly evaluated by the same physiotherapist who travelled to the different sites. If the principal physiotherapist was not available, the patients were evaluated by a physiotherapist trained and overseen by the principal physiotherapist. Data were controlled and monitored on a regular basis by the sponsor to achieve the same data quality as in a clinical trial. Additional patients have been recruited but were not included in the NHS data used here. Data were prospectively acquired using strict standard operating procedures and were systematically controlled. The study was approved by the relevant ethical review boards and by the French Health Authority and was registered on clinicaltrials.gov (NCT02057705). Its European extension was registered on clinicaltrials.gov under NCT03351270. Additional details are available in a previous publication [22].

**Table 1:** Number of patients in NatHis-CNM with *MTM1* and *DNM2* mutations by gender and age

	CNM mutation^a^	
	*MTM1*	*DNM2*
Gender		
Males	41	6
Females	3	9
Age (years)		
0–2	12	0
2–6	10	1
6–16	13	1
> 16	9	13

^a^No patients with *BIN1*-related CNM have been enrolled

## Results

### Adequacy of the disease progression model

The Bayesian methodology was applied to the domain of respiratory function, which is evaluated with different assessments (pulmonary function tests and time on ventilator) depending on the age and the respiratory status of the patient. FEV1 responses in children between 6 and 18 years of age are presented here. Although applicable to patients with *DNM2* or *MTM1* mutations, only the set of results for patients with the *MTM1* mutation are presented for sake of clarity. Other endpoint measures (time on ventilator for children and two assessments in the adult population, FEV1 and time on ventilator) are shown in additional figures. Additional analysis could be done on the collected motor item data; however, this will require a new set of multinomial modelling and will be done in future research.

**Fig. 1:**
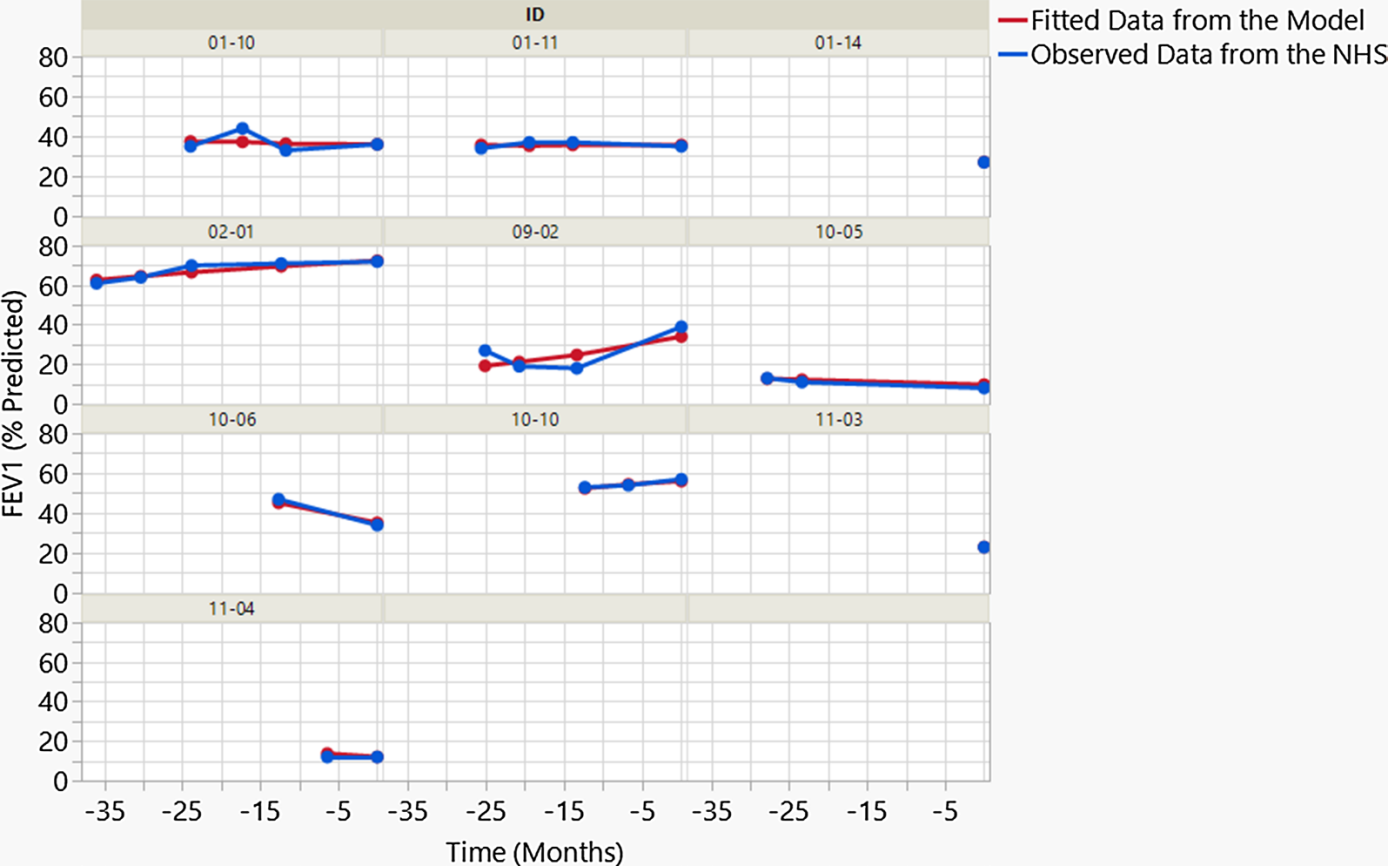
Adequacy of model fit for FEV1 (%) in children. Data for each subject is shown in blue. Model fit is shown in red

Based on the quality of fit, the model based on NHS data accurately describes the progression of the FEV1 scores with age in children (Fig. 1), FEV1 scores in adults (see Additional file 1), time on ventilator in children (see Additional file 2), and time on ventilator in adults (see Additional file 3).

### Predictive distribution and responder definition

**Fig. 2:**
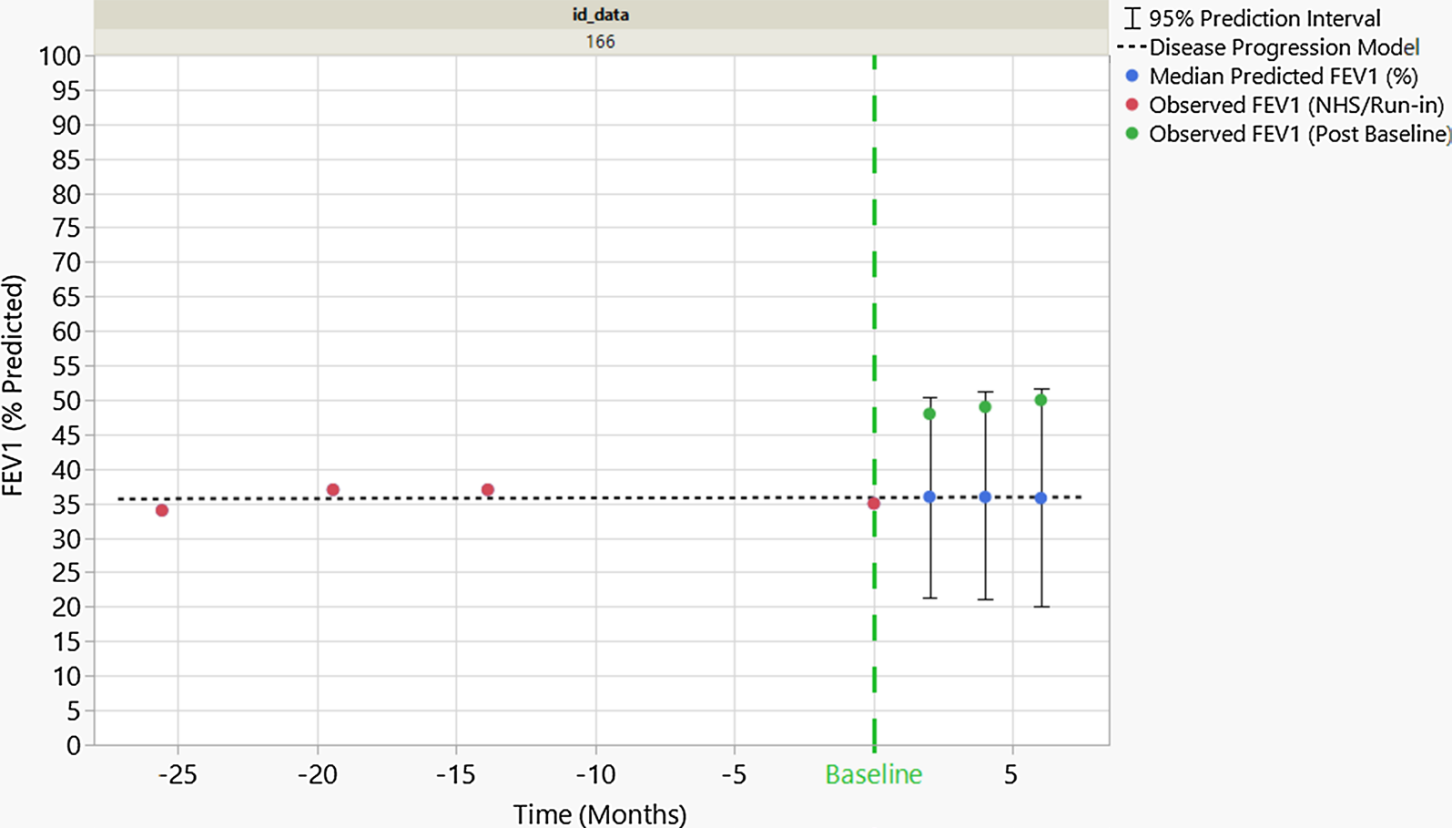
Predicted trajectory of FEV1 scores and actual scores for a patient with 26 months of NHS data. The conditional predictive distributions at 2, 4, and 6 months after treatment are represented using the black intervals. FEV1 scores observed during the study are in green. The individual FEV1 scores are within the 95% prediction intervals, but the joint probability to observe all 3 occurrences is less than 0.01

The Bayesian disease progression model as defined above can be used to define responders in a clinical trial. Using the Bayesian model, it is possible to derive the predictive distribution of the score values after treatment under the assumption that patients do not experience a treatment effect and continue their progression naturally as seen during the NHS. Figure 2 is a representative example of prediction of FEV1 score for one patient with 26 months of NHS data. Based on the observed NHS, a disease progression model is predicted (dotted line in Fig. 2). The joint probability using the (trivariate) predictive distribution can be computed based on actual measurements (green dots in Fig. 2). If the predictive probability of the measured improvements is smaller than 0.01 (as it is the case here, since the green dots lie in the upper tail of the predictive distribution), the patient is declared to be a responder. Our choice of a 0.01 predictive probability cut-off to define responders comes close to optimizing the design’s power. A threshold substantially lower than this would not allow us to detect treatment effects at all, whereas a higher (less stringent) threshold would detect many deviations, making it hard to distinguish truly responding patients from those who simply appear to “respond” due to high visit-to-visit variability in their trajectories.

**Fig. 3:**
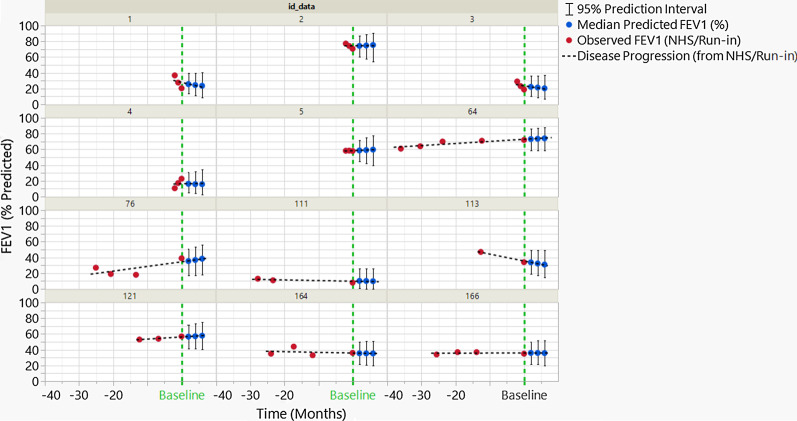
Distributions of FEV1 scores and predictions for seven patients from the NHS (2- and 3-digit identification numbers) and five patients for who only a run-in period data are available

In Fig. 3, the predictive distributions of the score values are shown for a simulated clinical trial including seven patients with NHS, and five patients for whom data are available only for a run-in period prior to treatment initiation. For the patients with NHS data, the predictions are rather consistent over the 6 months following the start of the treatment. However, for the patients with only run-in data (the first 5 patients in Fig. 3), the distribution of predicted data enlarges rapidly over time, reflecting that issue that limited historical data are available and prediction of evolution is unreliable. Although longer run-in periods produce a more stable projection, the influences of the number and timing of such points on the model’s predictive capabilities are marginal. There is also a modest effect of proximity to the upper or lower boundary on interval width (compare patients 111 and 113 in Fig. 3). Using this methodology, a patient is identified as a responder based on that individual’s predictive distribution, which accounts for the uncertainty in each individual’s disease progression.

Since response depends on each patient’s NHS, the endpoint measure (e.g., FEV1 or time on ventilator), and departure from their predicted disease progression, the responder statuses for all the patients can be combined into one single computation, independently of the age and endpoint measure used. Since data from patients with NHS will be combined with that from patients with only run-in data, there is a slight borrowing of information from the NHS patients to the run-in only patients. This borrowing is, however, moderate. The rate of responders and non-responders is the key endpoint for the trial design and influences its power, required sample size, and other key operating characteristics. There is no consensus to define what difference in FEV1 is clinically meaningful in a patient with CNM. Here, we considered 8% a clinically significant improvement for FEV1, since it is the annual rate of decline in teenagers with Duchenne muscular dystrophy [33]. The same approach could be conducted by defining another lower or higher threshold.

### Sample size computation

Using information about the number of responders from previous studies and assuming that the rate of response will follow a beta distribution, the conditional *assurance* (or expected power) of the posterior distribution of the rate of responders can be computed. Since there are no control groups, the power can be computed as the posterior probability that the rate of responders observed is greater than the rate of responders that would be observed assuming no change in the trajectory and using the observations drawn from the same predictive distribution.

Under the assumptions that each patient can act as their own control and that there will be no change in a patient’s trajectory without intervention, the rate of response that naturally occurs accounting for visit-to-visit variability can be assessed. By simulating a great number of studies with 12 patients and sampling individual values at the future visits, it is possible to determine if a patient is a responder or not. Note that these results are independent of the endpoint measure chosen. The rate of response will always have this distribution, as it is only linked to our definition of a responder, which is simply based on probabilistic reasoning (joint probabilities).

**Fig. 4:**
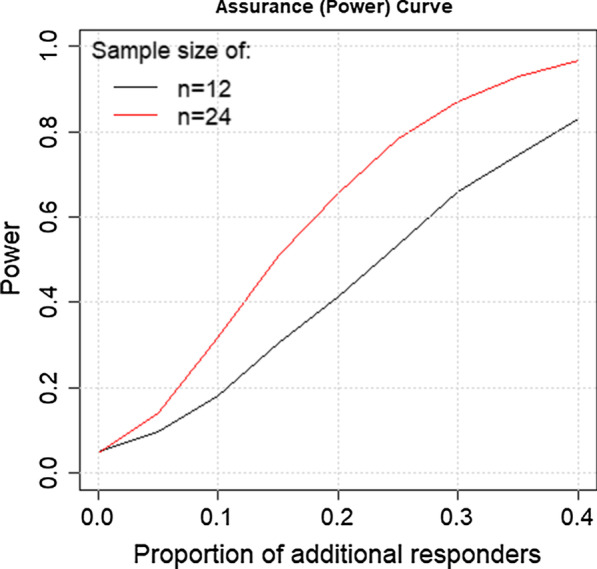
Assurance curve as a function of increase of rate of response for sample sizes of 12 (blue) and 24 (red)

In order to appropriately control the design’s Type 1 error, and due to the relatively small sample sizes, the probability by which the difference in responder rates needs to exceed zero must be adapted. In other words, to limit the probability of falsely declaring a treatment effect when a natural response is observed, the number of responders needs to exceed a certain threshold. Here, a threshold of 91% ensured a 5% type 1 error rate. Figure 4 shows an assurance curve as a function of the increase in the rate of response given a posterior distribution of the rate obtained from historical knowledge. When the true difference in the responder rates is 0, the Type I error is controlled at the traditional 0.05 level. Additional responders imply responders beyond those expected if left untreated. The horizontal axis corresponds to treatment effect, which is the difference in the proportion of responders in treated and untreated groups. The vertical axis gives the assurance (Bayesian power), which is the probability that the increase will be detected. Thus, an increase of 0 corresponds to the null hypothesis of no treatment effect, and the observed assurance of 0.05 at this value is the trial’s Type I error.

## Discussion

We have reported the application of Bayesian statistics to model the future natural history of a rare disease, centronuclear myopathy. To build this model, we used data from 4-year follow-up of 59 patients carrying mutations in the *MTM1* or *DNM2* genes. The model predicts individual patient trajectories for several endpoint measure scores based on the observations of a natural history study, and its quality of fit suggests that it adequately represents natural evolution of the disease.

Bayesian statistics offer the opportunity to compare the outcomes of patients at a given time after treatment to the simulated endpoint scores at the same given time without a treatment. Having predicted an individual trajectory with a certain probability allows us to estimate the probability that an observed deviation from that predicted trajectory would have happened without intervention. Consequently, our Bayesian incorporation of auxiliary data (NHS and run-in) offers an alternative to the comparison of treated patients with an untreated group, which can be challenging in small and heterogeneous cohorts, two conditions characteristic of clinical research in rare disease.

Bayesian statistics have previously been used in the field of rare diseases: Quintana et al. developed a Bayesian model of disease progression in GNE myopathy based on quantitative muscle strength data [34]. Ramanan et al. proposed a Bayesian design for a phase 2 trial to compare adalimumab versus pamidronate in chronic nonbacterial osteomyelitis [35]. In the currently ongoing Sarcome-13 trial, a phase 2 trial of mifamurtide in newly diagnosed high-risk osteosarcoma, a Bayesian analysis is planned that will incorporate available historical data into the trial [36]. A review of Bayesian methods in rare disease settings has recently been published [37].

The natural evolution of CNMs consists of an overall stability of the patients’ parameters [28], and, consistently with this, the model shows non-progression of the disease. Therefore, this type of analysis will not identify a stabilising effect in centronuclear myopathy. This will be true whatever the design of the trial. As long as the conditions of the patients do not deteriorate over the period of time during which a trial can be organised and completed (generally 2 years), a long-term stabilizing effect of the treatment will not be possible to demonstrate.

One of the main limitations of our study is that it relies on a restricted and heterogeneous sample size. However, this small and heterogeneous sample size is actually the driving rationale of the Bayesian approach as small sample sizes are typical in the fields of rare diseases [38, 39]. In the field of centronuclear myopathy, the study described is by far the largest prospective cohort to date. Indeed, existing studies that we found on the natural history, genotype, and phenotype of patients living with centronuclear myopathy that used larger cohorts of up to 120 patients were mostly retrospective or involved only a punctual intervention to identify mutations [40–42].

An additional limitation resides in the heterogeneous follow-up. Some patients were followed for 1 year, some for 4 years. The model could be used for a therapeutic trial with an even more limited follow-up, however, as clinical trials generally last about 1 year. Therefore, not all of the model’s trajectories derive from the observed results, but the trajectories of the run-in patients borrow information from the natural history study of patients with longer follow up times. Again, this limitation is intrinsic to the nature of a very rare disease. The inclusion of all patients at the same time is extremely challenging in part due to geography—the whole of Europe in the present study. As the borrowing of data is limited and the Type I error is well controlled, this borrowing is justified. The Type I error is the rejection of a true null hypothesis (i.e., a false positive). In this case, a Type I error results in the conclusion that a treatment is effective when the patient’s response is in fact natural, and apparent improvement is due merely to visit-to-visit variability. To limit this, our method adapts the threshold difference between the predicted and observed response rates that is required to declare a treatment effect.

Because the natural evolution of the patients is not necessarily linearly correlated to previous history, the reliability of the model’s prediction will decrease with time. As is shown by the comparison of patients with considerable NHS data to those with run-in data, the more data available, the more stable the predictive distribution. Although the reliability of the model is therefore limited by the duration of the natural history study, the same limitation is found in a randomised controlled trial that can only show treatment efficiency for the duration of the trial. We acknowledge that by avoiding randomization and blind assessment in order to minimize sample size, we are forfeiting some protection against possible systemic biases that could result from assumption that the natural evolution is linear, and the possibility of a placebo effect. In particular, the study’s Type I error and power will obviously be affected by model misspecification. Manifestations of a placebo effect could include a positive adjustment to the intercept starting at the time of the intervention, a slight increase in slope, or even a departure from linearity (all on the logistic scale). Investigations of such changes and their impacts were discussed in the recent poster presentation by Monseur et al. [43] and will be the subject of a future manuscript.

Though it is currently unclear how large the placebo effect can be in patients with CNMs, the placebo effect in other neuromuscular disorders has been described as mild and transient for spinal muscular atrophy and as non-existent for Duchenne muscular dystrophy [44]. The placebo effect observed in double-blind placebo-controlled studies in spinal muscular atrophy patients, for instance, is limited in duration to up to 6 months [45]. In recent trials involving Duchenne muscular dystrophy patients, it has been demonstrated that natural history study data are highly comparable to data from patients treated with placebo [44]. The quality of fit of the model shows an adequate prediction of evolution over the time of the NHS. Therefore, it can be assumed that, during that clinical trial period, variations in evolution compared to the natural history predictions will be due to a treatment effect rather than a lack of reliability of the model. However, the model does not predict rare events, such as a lower respiratory tract infection that would require a hospitalisation and that could induce a significant functional decline. Mitigating this, the probability of these rare events is known. In a prospective study of a 33-patient cohort over a 1-year period, 17 (52%) patients required a visit to the hospital for acute care, with a total of 38 visits (1.15 annual visit rate). Of visits to the emergency room, 47% were due to fever or infection, and of the 34% that resulted in hospitalisation, 69% were due to fever or infection [29].

Bayesian incorporation of auxiliary data can reduce the number of patients necessary to conduct a study and the number of patients who must be given placebo, but cannot deliver a conclusion with the same level of evidence as a full two-arm blinded study. Indeed, the model is constructed on the basis of NHS data in which no placebo effect is expected—from either the patient or from the evaluator perspective—and the evolution of patients after a given intervention could differ from the predicted trajectory due to a placebo effect. Having a limited number of patients on placebo or progressively switching patients from placebo to active treatment may overcome or mitigate this issue. In addition, a limited number of placebo-treated patients could also help to verify that untreated patients actually follow model predictions. A similar design is being used in Audentes’ ASPIRO trial, an open-label trial for gene transfer therapy in XLMTM (NCT03199469). In the extension phase of this trial, subjects who were in the delayed treatment control group are administered the drug on trial after having completed their last visit as a control at Week 24, when the primary efficacy endpoint measures will have been assessed.

As in any clinical trial occurring in a very heterogeneous and rare population, selection biases may arise. It is important to note that, in order to minimise biases in the present study, all the centres were contacted when recruiting participants and a single physiotherapist was hired to travel and visit the European patients. Social bias was also avoided by covering the patients’ costs. Furthermore, data heterogeneity is already very large with patients covering the whole spectra of possible values on the scale under investigation. Although the risk of bias cannot be entirely mitigated, no fundamental differences are expected between the patients currently enrolled and those not.

The aim of Bayesian approach is not to lower the level of evidence required for drug approval in rare diseases, but rather to benchmark this level as close as possible to the one of drug development in more common diseases, taking into account the limited existing population and the heterogeneity in terms of age, genotype, and severity. In using an individual patient’s trajectory and borrowing information on day-to-day variability from the population to more reliably predict the individual’s course of disease and then defining a response as deviation from this course, our model can estimate treatment efficacy across patients with different disease severities. Demonstrating efficacy, even if moderate, in post-symptomatic patients may also justify moving to younger or pre-symptomatic patients where the effect can be much more dramatic, given the better state of the targeted tissue. This has been clearly demonstrated in spinal muscular atrophy [46]: The effect demonstrated in a double-blind placebo controlled study in post-symptomatic patients [47] was much more dramatic in a pre-symptomatic population [48], leading to newborn screening programs across the world [49] and a dramatic improvement in patients’ conditions. Similarly, the ability to demonstrate even a mild effect in a post-symptomatic population where an analysis between treated and placebo-controlled patients cannot be conducted for practical reasons can provide evidence supporting use of a therapy in a younger or pre-symptomatic population that cannot be initially targeted by clinical development but who are likely to benefit the most and who are likely to have the best benefit to cost ratio from a payer perspective [50].

## Conclusions

In summary, the major and innovative idea described here is to use Bayesian statistics in the development of disease progression models to construct individual-level predictive distributions to identify the time point at which a patient’s response is expected. This type of analysis allows reliable simulation of the evolution of a patient’s response trajectory and comparison to observed outcomes after treatment, thus bypassing the issue of small and heterogeneous patient cohorts typical of clinical trials in rare disease populations.

Bayesian statistics represent a methodologically valid and attractive option in the field of rare diseases that is increasingly accepted by regulatory authorities. However, we are not aware of any medication that has been approved based on a pivotal study conducted using a Bayesian natural history model. Future peer-reviewed publications of data from therapeutic trials that rely on this type of modelling as well as ongoing dialog with regulatory authorities will contribute to the broader use of this approach with consequent benefits to the lives of persons struggling with rare disease.

## Supplementary Information

**Additional file 1:** FEV1 (%) in adults. Data for each subject is shown in blue. Model fit is shown in red..

**Additional file 2:** Time on ventilator in children. Data for each subject is shown in blue. Model fit is shown in red.

**Additional file 3:** Time on ventilator in adults. Data for each subject is shown in blue. Model fit is shown in red.

## Data Availability

All data published here are available upon request to the corresponding author (LS).

## Abbreviations

CNM: centronuclear myopathy
EMA: European Medicines Agency
FDA: U.S. Food and Drug Administration
FEV1: forced expiratory volume in 1 s
NHS: natural history study
XLMTM: X-linked myotubular myopathy
