# Supplementary figures and images for: Hierarchical Bayesian modelling of disease progression to inform clinical trial design in centronuclear myopathy
Source: Orphanet J Rare Dis. 2021 Jan 6;16:3. doi: 10.1186/s13023-020-01663-7 (PMC7789189; doi:10.1186/s13023-020-01663-7)

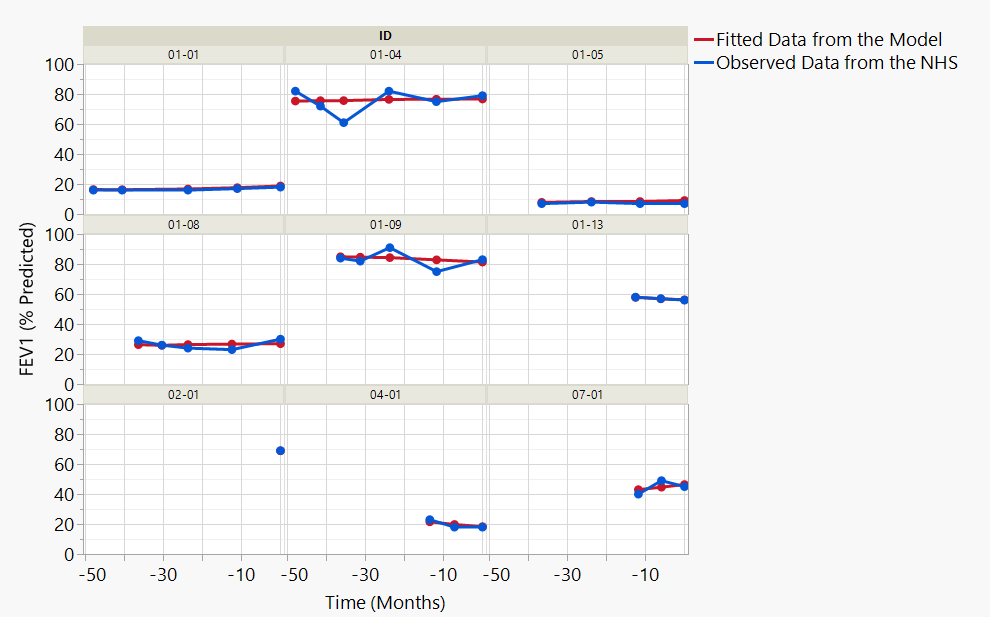

Supplement: Supplementary file 1 — Additional file 1: FEV1 (%) in adults. Data for each subject is shown in blue. Model fit is shown in red.. [file 13023_2020_1663_MOESM1_ESM.png]

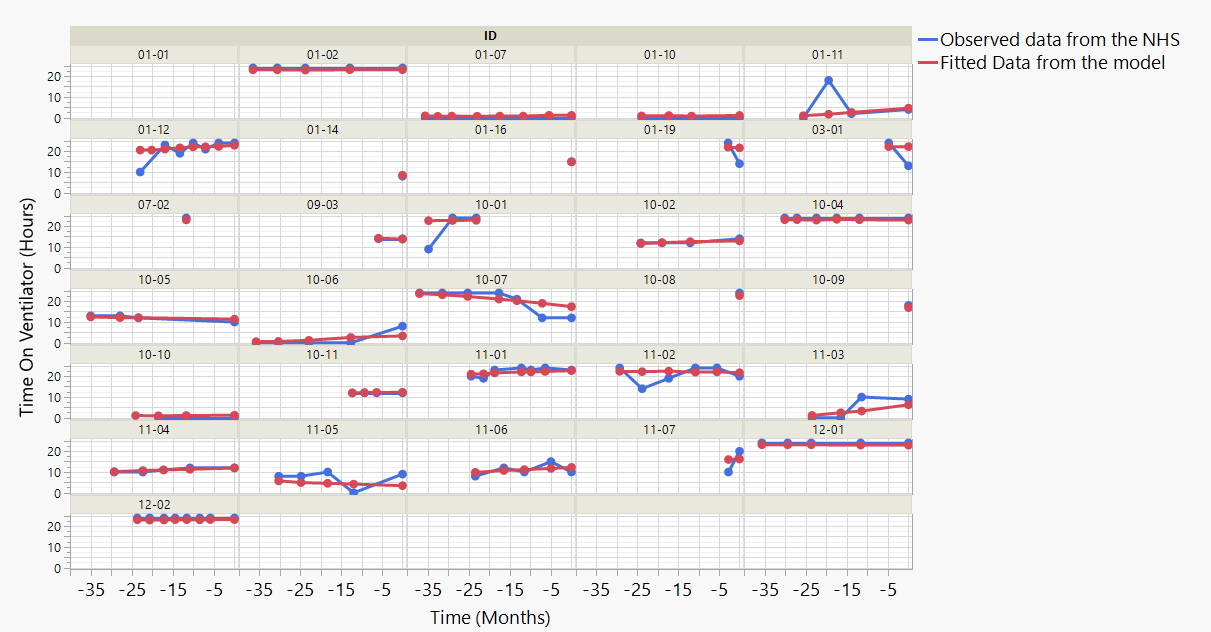

Supplement: Supplementary file 2 — Additional file 2: Time on ventilator in children. Data for each subject is shown in blue. Model fit is shown in red. [file 13023_2020_1663_MOESM2_ESM.png]

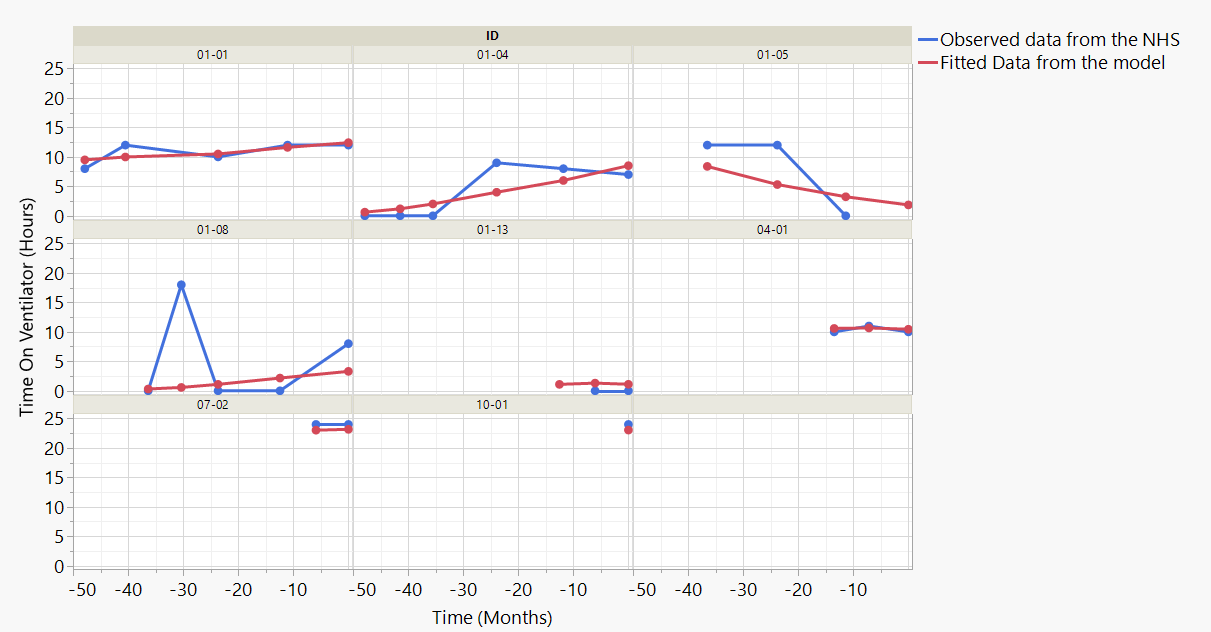

Supplement: Supplementary file 3 — Additional file 3: Time on ventilator in adults. Data for each subject is shown in blue. Model fit is shown in red. [file 13023_2020_1663_MOESM3_ESM.png]
